# Brain Ventricle and Choroid Plexus Morphology as Predictor of Treatment Response: Findings from the EMBARC Study

**DOI:** 10.21203/rs.3.rs-2618151/v1

**Published:** 2023-03-01

**Authors:** Harald Murck, Maurizio Fava, Cristina Cusin, Cherise Chin Fatt, Madhukar Trivedi

**Affiliations:** 1. Dept. of Psychiatry and Psychotherapy, Philipps-University Marburg, Marburg, Germany; 2. Department of Psychiatry, Massachusetts General Hospital, Harvard Medical School, Boston, MA, USA; 3. The University of Texas Southwestern Medical Center, Department of Psychiatry, Center for Depression Research and Clinical Care, Department of Psychiatry, Dallas, USA

## Abstract

Recent observations suggest a role of the choroid plexus (CP) and cerebral ventricle volume (CV), to identify treatment resistance of major depressive disorder (MDD). We tested the hypothesis that these markers are associated with clinical improvement in subjects from the EMBARC study, as implied by a recent pilot study. The EMBARC study characterized biological markers in a randomized placebo-controlled trial of sertraline vs. placebo in patients with MDD. Association of baseline volumes of CV, CP and of the corpus callosum (CC) with treatment response after 4 weeks treatment were evaluated. 171 subjects (61 male, 110 female) completed the 4 week assessments; gender, site and age were taken into account for this analyses. As previously reported, no treatment effect of sertraline was observed, but prognostic markers for clinical improvement were identified. Responders (n = 54) had significantly smaller volumes of the CP and lateral ventricles, whereas the volume of mid-anterior and mid-posterior CC was significantly larger compared to non-responders (n = 117). A positive correlation between CV volume and CP volume was observed, whereas a negative correlation between CV volume and both central-anterior and central-posterior parts of the CC emerged. In an exploratory way correlations between enlarged VV and CP volume on the one hand and signs of metabolic syndrome, in particular triglyceride plasma concentrations, were observed. A primary abnormality of CP function in MDD may be associated with increased ventricles, compression of white matter volume, which may affect treatment response speed or outcome. Metabolic markers may mediate this relationship.

## Introduction

The pathophysiology of major depressive disorder (MDD) is heterogeneous. The identification of effective compounds on the basis of a specific underlying neurobiology is hampered by the currently accepted definition of MDD in relevant classifications, including the DSM-5, which does not take biological differentiation into account. Importantly, this variability may not only affect the response to a given pharmacotherapy, but also the natural course of clinical change. This situation has negative implications in the context of clinical trials, in which treatment arms are compared, which may show neurobiological heterogeneity at baseline. To stratify a population on the basis of biological variables would confirm a biologically defined subtype, which is suitable for the treatment of a specific nature.

An argument, which is often brought up as a challenge is the operational complexity of such an approach. However, broadly available and easily accessible biological markers are available. Markers, which are available and have been shown to differentiate patients with depression include inflammatory markers^1–5^, metabolic markers, in particular those related to metabolic syndrome^5, 6^, and neuroendocrine^1, 7, 8^ characteristics. More recently, markers of autonomic regulation, including blood pressure and heart rate variability received renewed attention^9–12^.

Furthermore, imaging biomarkers have been characterized to differentiate the subjects with presumed different clinical response. Many of these, including volumetry of gray- or white matter segments are of high importance from a research perspective, but are difficult to assess in standard practice^13, 14^. A more easily accessible imaging marker, which is unfortunately frequently not reported in recent imaging studies, is cerebral ventricular volume (VV), partially by the argument that changes in ventricular volume are biologically unspecific, as many different brain areas may contribute to this phenomenon. Here we explore the alternative hypothesis that choroid plexus driven ventricular expansion results in the compression surrounding anatomical areas, making ventricular volume changes the potential primary factor. In the context of depression, VV is increased in patients with depression in comparison to healthy controls^15–17^ and may be related to treatment outcome^18^. We recently demonstrated an association between an increased choroid plexus and ventricular volume and worse treatment outcome in hospitalized patients with depression and identified moderators of this relationship^19^, i.e. body mass index (BMI) and the salivary aldosterone/cortisol ratio. The effect may be mediated by a compression of of corpus callosum segments, which will affect anatomical projection areas.

In this context it is important to consider that VV and the volume of the corpus callosum show short term structural plasticity. Both underly sleep-related changes^20^ and VV is sensitive to stress, at least in animals^21^. A plausible mediator of these phenomena is again the change in activity of the choroid plexus (CP). The volumetric determination of the CP is a relatively new area of investigation, but is feasible with current MRI techniques. Changes have been described in complex pain syndrome^22^, anorexia nervosa^23^, multiple sclerosis^24^ and most recently in major depression^25^ and psychosis^26^. Mechanistically, stress leads in an animal model to changes in gene expression of the CP of receptors, which have been linked to MDD, including 5-HT2a, 5-HT2c, glucocorticoid, TNFα, IL1β, BDNF^27^ as well as IL1 receptor^28^ and the CRH-receptor^29^. The choroid plexus may play a role in inducing inflammatory changes in depression and may be involved in sickness behavior^2^. Downstream mechanisms of the involvement of the CP are therefore at least twofold: an increased CSF release may lead to a mechanical compression of anatomical areas, which are adjacent to the ventricles^30^. Secondly, molecular moderators may spread into brain tissue via volume transmission^31, 32^. Those moderators may be produced by the CP itself or stem from the circulation.

We want to replicate our earlier findings of the relationship between clinical outcome of patients with depression on the one hand and ventricular volume, choroid plexus function and corpus callosum volume in a larger sample in this retrospective analysis from data from the EMBARC study. In an exploratory way we also correlate metabolic and autonomic markers with the volume of these anatomical areas in order to generate hypothesis of the causality of the observed relationships.

## Methods

The EMBARC study characterized biological markers in a randomized placebo-controlled trial of sertraline vs. placebo in patients with MDD for 8 weeks, followed by an additional treatment section, based on the outcome of the first 8 weeks of treatment. For consort statement see^33^. This trial is conducted according to the Declaration of Helsinki. It was approved by the Institutional Review Board at each clinical site. Signed informed consent was obtained from subjects in order to participate in the trial. The main objective was to identify clinical and biological moderators of treatment response^34^. Patients with early onset (before age 30), chronicity (episode duration > 2 years) or recurrent MDD (two or more recurrences including current episode) were enrolled.

The clinical parameter of interest was the Hamilton-depression rating scale (17 item; HAMD-17). For correlational analysis of clinical improvement we used the ratio between the HAMD-17 at outcome divided to the HAMD-17 at baseline (HAMD-17 ratio). A value of 1 means no change from baseline, a value of 0.7 means a reduction to 70% of the baseline value. Response was defined as a HAMD-17 ratio ≤ 0.5.

For these primary analyses we focused on subjects, who completed the first 4 weeks of the placebo-controlled treatment phase. In the current dataset, 207 subjects had assessments with the Hamilton depression rating scale (HAMD) at baseline, of which 171 (; age 37.5 ± 13.4; HAMD-17: 18.8 ±4.7) had an assessment at week 4. We a priori chose the 4-week treatment interval in order to optimize the time for clinical improvement with the number of drop-outs. For a time course of the correlation of the HAMD-17 value with imaging parameters, which we generated as a sensitivity analysis and to show consistency, please see Table S1.

Imaging was processed as described before^34, 35^. Of the subjects, who completed 4 weeks of treatment, 171 also had imaging data at baseline. Association of volumes of CV, CP and of the corpus callosum (CC), with treatment response were evaluated. The relationship of the volume of choroid plexus, cerebral ventricular volumes and the corpus callosum with response after 4 weeks from baseline (≤50 % reduction of the HAMD) was assessed; gender, age, and total brain volume were taken into account for the primary MANCOVA analysis. For the analysis of correlations Pearson correlation coefficients and p-values are provided. The relationship between the volumes of the choid plexus- and ventricular volumes should be regarded as primary analysis, as this analysis serves to replicate our earlier findings. As the anatomical parameters of interest are considered to be highly correlated and therefore not independent correction for multiple testing was not performed. The correlations with metabolic and autonomic parameters have to be regarded as exploratory.

## Results

A correlation between baseline HAMD-17 and the volumes of interest was performed in order to determine potential state related effects. Choroid plexus volumes were significantly correlated with the HAMD-17 score at baseline (n = 217; right: Pearson R: 0.22, p = 0.002; left: Pearson R = 0.17, p = 0.017), whereas no correlation between ventricular volumes or corpus callosum sections and baseline depression severity could be detected (all p > 0.20 with the exception of the right lateral ventricle, which showed a trend toward a significant correlation (Pearson R = 0.13; p = 0.06).

Regarding the analysis of factors related to treatment outcome: No statistically significant treatment effect of sertraline was observed, as reported earlier^36^, but prognostic markers for therapy response were identified. Therefore, treatment was not a factor of the analyses. Comparing responders and non-responders, we adjusted for gender and age. An overall global significant difference between responders and non-responders was observed for volumetric parameters (p = 0.007, see table 2). Univariate analyses revealed that responders at week 4 had significantly smaller volumes of the choroid plexi and lateral ventricles, whereas the volume of mid-anterior and mid-posterior CC was significantly larger compared to non-responders (Table 2).

**Figure 1:**
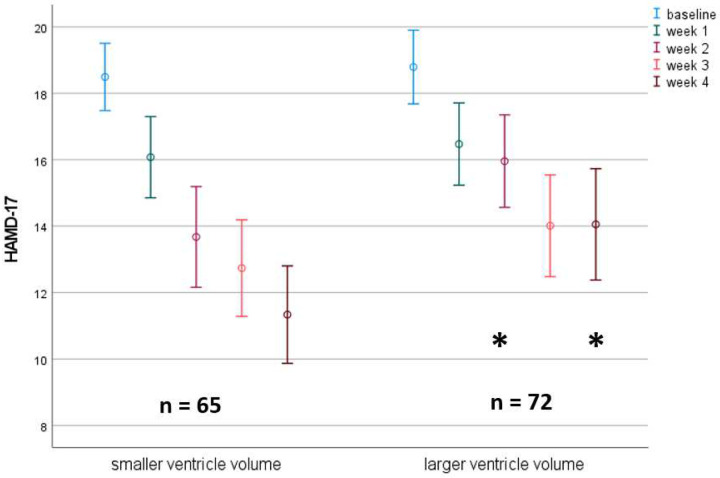
Time course of HAMD-17 score in subjects with larger vs. smaller right lateral ventricle volume. A median split was used to separate the groups. Only subjects without missing values are depicted.

Vice versa, splitting the population at the median for the ventricular volume demonstrates the difference of the course of depressive symptoms between the two VV groups clearly (Fig. 1): A significant difference between HAMD-17 scores for the high vs. low VV-volume groups were observed at week 2 and week 4.

As a sensitivity analysis we also compared the anatomical structures split into responders vs. non-responders for each timepoint of the study, up to 8 weeks. Choroid plexus volumes at baseline differentiated these groups starting at week 4 up to week 8 (p < 0.05), however, other parameters did not reach statistical significance past week 4. Please see suppl. Table S1 for the stability of the correlation between volume of anatomical structures and treatment effect over time.

**Figure 2:**
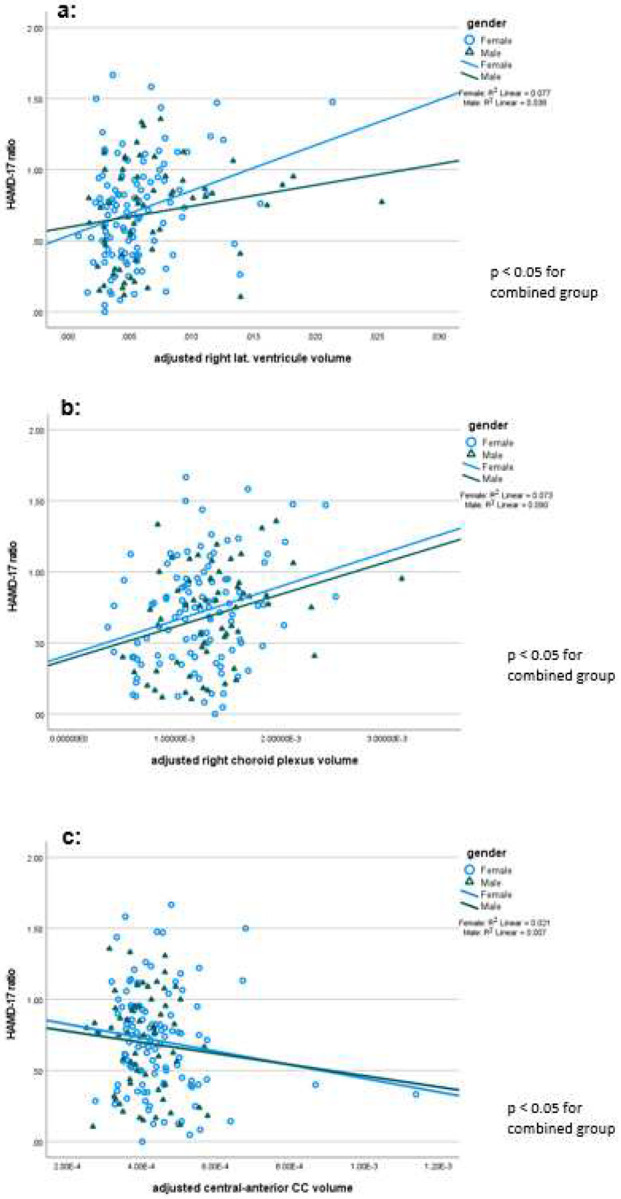
a) Correlation of HAMD-17 by right lateral ventricle volume: a larger ventricle volume is associated with less favorable clinical improvement after 4 weeks, independent of gender. b: Correlation of HAMD-17 by right lateral ventricle volume: A choroid plexus volume is associated with less favorable clinical improvement after 4 weeks, independent of gender. c: Correlation of HAMD-17 by central anterior CC volume ratio: A smaller CC volume is associated with less favorable clinical improvement after 4 weeks, independent of gender.

In addition to the comparisons between responders and non-responders correlations between baseline parameters and the HAMD-17 ratio were performed, which is independent of a chosen cut off. These correlational analyses confirmed the relationship between clinical change on one hand and ventricular volume, choroid plexus volume and CC segment volumes at baseline on the other hand (Tab. 3, Fig. 2). These data as well as the previous ones confirm the difference between responders and non-responders regarding anatomical structures and therefore the results from our pilot study. In addition, we explored other factors, which may affect the volume of the anatomical arias in an exploratory fashion. These analyses, are part of Tab.3 and need replication. We found that the volumes of both lateral ventricles were positively correlated to LDL-cholesterol and triglyceride levels. The volume of the left VV was significantly correlated and the right VV showed a trend towards a significant correlation to systolic blood pressure. A similar pattern was observed for the CP volumes. All these parameters are also positively correlated to age, which we corrected for in the primary analysis.

In order to determine the relationship between the anatomical areas of interest, ventricular volumes were correlated with CC segments and CP volumes. A significant positive correlation between CP volumes and lateral ventricle volumes was established. More importantly, a significant negative correlation between third ventricular volumes and the mid-anterior and mid-posterior CC segments, as well as a significant negative correlation between the lateral ventricles and the mid-posterior CC volume were observed (Table S2).

DTI parameters as assessed for the corpus callosum did not predict outcome. However, the volume of the mid-anterior and mid posterior CC segments, adjusted for total brain volume, correlated negatively with the axial diffusivity of these segments (mid-anterior: R = −0.42, p < 0.001, n = 191; mid-posterior: R=−0.15; p =0.036, n = 196), whereas the CC-segment volumes were not associated with fractional anisotropy (for all, p > 0.1).

## Discussion

The primary outcome of this study is that an easily accessible imaging marker, i.e. lateral ventricular volumes, show a strong predictive value for the improvement of depressive symptoms in MDD patients treated with either sertraline or placebo. Mechanistically, this appears to be related to an alteration in choroid plexus function, both of which may affect corpus callosum integrity.

The strong relationship of ventricular volume to choroid plexus volume on one hand and the volume of CC segments on the other hand could be of theoretical interest for the pathophysiology of some forms of MDD. A working hypothesis could be that changes in choroid plexus function, i.e. an increased release of CSF volume^19, 37^, or an increased release of specific bioactive molecules, including inflammation mediators^24, 31^, may lead to a change in white matter volume and/or integrity. The increased ventricular volume or, alternatively, such bioactive molecules may affect white matter function either by mechanical compression or an effect on white matter integrity via alternations of oligodendrocyte function. This could be related to changes in myelination or changes in the volume regulation of axons within the CC. Disturbance of white matter integrity has indeed frequently been described in patients with depressive disorders, mainly by using diffusion tensor imaging (DTI) methods^38–43^. In support of the hypothesis of the choroid plexus involvement in this pathway: the activity of the choroid plexus is affected by neuroendocrine influences, which have been linked to MDD, in particular vasopressin and aldosterone^19, 37^, as well as metabolic markers related to an increased BMI^19, 44^. These findings are also in line with the role of inflammation as both aldosterone^45–47^ and high BMI^48–51^ show a close association to increased inflammation. Finally, inflammation has recently been associated with increased choroid plexus volume in patients with depression^25^ and multiple sclerosis^24^.

Our observation that the HDRS-17 score correlates significantly to choroid plexus volumes at baseline, only by trend to ventricular volumes and not to CC segment volumes implies that choroid plexus volume shows a state characteristic, and that ventricular volume shows somewhat lesser plasticity in relationship to mood and may have a more trait/chronicity related characteristic. Corpus callosum segments volume furthermore appear mainly to be trait- or risk markers. As mentioned in the introduction, stress leads to an increase in ventricular volume in animals^21^. Childhood abuse has been related to later life increase ventricular volumes and reduced white matter volume^52, 53^ and also the therapy refractoriness in depression^54, 55^. This could imply that early life stress affects ventricular and white matter structure via a prolonged choroid plexus activation. However, a recent analysis, based on the self-report depression scale QIDS-SR did not confirm a difference regarding subjects with and without childhood adversity regarding clinical response^56^.

Other factors, which determine the size of the ventricles, which are potentially mediated via choroid plexus alterations are related to metabolic disorders. In particular, high fat diet is related to an increased ventricular volume in animals in the context of traumatic stress^57^. The finding of the correlation between triglyceride levels and systolic blood pressure at baseline on one hand and choroid plexus volumes and ventricular volumes on the other hand reported here confirm the influence of metabolic parameters to differentiate patients with depression^1^. Interestingly, and a link between increased ventricular volume and metabolic dysfunction, in particular hyperlipidemia, in subjects with normal pressure hydrocephalus^58^ has also been observed. Similarly, in our pilot study we previously described a strong correlation between BMI and both choroid plexus- and ventricular volume^19^.

As mentioned, markers of inflammation and metabolic disturbances are preferentially present in subjects with atypical depression, in comparison both to healthy subjects and patients with melancholic depression^1, 59, 60^. This is in line with the current findings, as atypical depression appears to be less responsive to standard antidepressant treatment^61^. Of importance, patients with atypical depression show in general an earlier age of onset^62^. The current study only enrolled patients with an age of onset ≤ 30 years, which means that there is probably an enrichment of this subtype in comparison to the general population. Age of onset appears to be associated with specific neurobiological differences in depression^63^, which may be related to alterations in autonomic function and endocrine characteristics. Whether age of onset also differentiates brain morphology needs further confirmation.

Regarding the relationship of DTI parameters, no relationship with clinical change was observed. This is in contrast to studies, which reported DTI parameters as predictive for response, for example to ketamine^64, 65^. Nevertheless, we observed that the volume of CC segments correlated inversely with axial diffusivity (AD), i.e. a smaller CC segment volume was correlated to an increased AD. An earlier DTI report from the EMBARC study, which focused on the structural connectivity in specific anatomical areas did find an increase in fractional anisotropy (FA) in non-remitters^35^. As AD and FA are correlated, this outcome appears consistent, but is nevertheless in contrast to a number of earlier cited findings^39, 40, 42, 43, 66^. This shows the importance to take into consideration that FA and AD and other DTI markers can be influenced by varying mechanisms, which depend on one hand on the structural integrity of an axon, but also an axonal density^67^.

Limitations of the study are the post hoc nature of the current analyses, however, they were motivated by the attempt to replicate data from an earlier study^19^ and the primary variables of interest are identical. Therefore, with all caution, the current analysis overall confirms the previous pilot study. It has, however, to be considered that inclusion/exclusion criteria differ between the studies.

In conclusion, we (re-)identified an easily accessible imaging marker which appears to be related to the clinical course of depression. Ventricular volume may affect other imaging parameters and should therefore be taken into account in future imaging studies, at least in studies in MDD. In addition, the current findings go beyond a strictly descriptive association. With the additional observation of the relationship of increased ventricular volumes and increased choroid plexus volumes, our findings provide a plausible hypothesis, how neuroendocrine and metabolic parameters mechanistically influence depressive symptoms. A new focus on choroid plexus function in stress-related disorders appears to be supported.
